# A Preventive Social Media Intervention for Perinatal Depression and Anxiety in Regional, Rural, and Remote Communities: Participatory Co-Design Study

**DOI:** 10.2196/91778

**Published:** 2026-06-10

**Authors:** Kacey Jane Lynch, Adrian Brian Royce Shatte, Jessica Muller, Kendall George, Gisele Rossini, Angela Anson, Courtney Hala, James Dimmock, Delyse Hutchinson, Samantha Teague

**Affiliations:** 1 Department of Psychology, College of Healthcare Sciences, James Cook University, 1 James Cook Drive, Townsville, Queensland, 4814, Australia, 61 74781 6354; 2 Department of Information Technology, College of Science and Engineering, James Cook University, Townsville, Queensland, Australia; 3 Margaret Roderick Centre for Mental Health Research, James Cook University, Townsville, Queensland, Australia; 4 National Centre for Indigenous Genomics, Australian National University, Canberra, Australian Capital Territory, Australia; 5 Department of Nursing and Midwifery, College of Healthcare Sciences, James Cook University, Townsville, Queensland, Australia; 6 Women's and Children's Service Group, Townsville Hospital, Townsville, Queensland, Australia; 7 Cairns Perinatal and Infant Mental Health (PIMH), Cairns Hospital, Cairns, Queensland, Australia; 8 Children’s Health Queensland Hospital and Health Service, Brisbane, Queensland, Australia; 9 Deakin Lifespan Institute, School of Psychology, Faculty of Health, Deakin University, Melbourne, Australia; 10 Centre for Adolescent Health, Murdoch Childrens Research Institute, Melbourne Royal Childrens Hospital, Melbourne, Australia; 11 Department of Paediatrics, University of Melbourne, Melbourne, Australia; 12 National Drug and Alcohol Research Centre, University of New South Wales, Sydney, Australia

**Keywords:** prevention, mental health, perinatal, maternal, depression, anxiety, pregnant, regional, rural, remote, social media, telemedicine, participatory research, co-design, patient and public involvement, PPI

## Abstract

**Background:**

Perinatal depression and anxiety are significant public health concerns, affecting up to 1 in 5 women globally, with disproportionate burden carried by women in regional, rural, and remote communities where structural and social inequities amplify vulnerability. Access to perinatal mental health support in these settings is severely constrained by geographical isolation, workforce shortages, financial barriers, and a lack of culturally safe services. Prevention is recognized as critical to reducing this burden, with evidence suggesting that effective preventive approaches can reduce population-level illness by up to 40% and alleviate downstream demand on overstretched services. Digital mental health interventions hold promise for improving access to support, yet few are co-designed with underserved perinatal populations.

**Objective:**

This study aimed to identify the mental health needs of perinatal women in regional, rural, and remote communities and to co-design a framework for a preventive social media–based intervention informed by platform-specific affordances and constraints, using Northern Queensland, Australia, as an exemplar.

**Methods:**

Using a participatory co-design approach, 26 perinatal women (21 postnatal and 5 antenatal) and 8 mental health care professionals from regional, rural, and remote Northern Queensland participated in focus groups or interviews, supplemented by ongoing consultation with a community advisory group comprising lived experience representatives, clinicians, and local community leaders. Qualitative data were analyzed using reflexive thematic analysis to identify core community mental health needs. Identified needs were then examined through a needs-affordances framework to determine how specific platform features could address, enable, or constrain those needs in the context of a preventive intervention.

**Results:**

Five core mental health needs were identified: (1) social connection and support; (2) personalized and respectful health care; (3) information that empowers; (4) place-based and culturally safe support; and (5) accessible, low-burden digital formats. Participants viewed social media as a potentially useful platform for fostering peer connection, normalizing perinatal experiences, and providing timely psychoeducation. However, both mothers and professionals expressed concerns about misinformation, harmful social comparison, and privacy risks that must be proactively addressed in program design. These insights were synthesized into a set of prototype design guidelines specifying recommended content, features, tone, and delivery formats to inform subsequent intervention development.

**Conclusions:**

This study provides a place-based, co-designed needs-affordances framework to guide the development of a preventive social media–based intervention for perinatal mental health support in regional, rural, and remote communities. The findings demonstrate that social media is an acceptable and promising platform for preventive perinatal mental health support in these settings, provided that design is driven by community need, platform affordances are systematically analyzed, and known risks are explicitly mitigated. These findings address a significant gap in the literature and offer a replicable methodological approach for co-designing contextually relevant digital mental health interventions with underserved populations.

## Introduction

Perinatal depression and anxiety are major public health concerns globally, affecting approximately 1 in 5 women across pregnancy and the first postpartum year, with true prevalence likely higher due to stigma and underreporting [1,2]. These conditions impair maternal functioning, strain intimate and family relationships, and reduce women’s quality of life [1,3]. Impacts also extend to infants, who face increased risks of poorer birth outcomes, developmental delays, behavioral dysregulation, and long-term social-emotional difficulties [4].

Australia’s public health care system, Medicare, provides universal access to a broad range of medical services through a hybrid public-private model; however, the practical availability of specialized mental health and perinatal care varies enormously by geography [5]. In Queensland, perinatal mental health care services are primarily delivered through public maternity hospitals, community health care centers, and primary care providers under the coordination of primary health networks, such as the Northern Queensland primary health network, which covers a vast and geographically dispersed catchment area spanning from St Lawrence to the Torres Strait and west to Croydon and Kowanyama [6]. This region is characterized by small and remote communities, higher proportions of First Nations populations, and a critical shortage of specialist mental health and perinatal services relative to need [6].

Access to timely and culturally appropriate support is therefore particularly limited in regional, rural, and remote communities across this area, where structural inequities create substantial barriers to care, including geographical isolation, workforce shortages, financial strain, and limited specialist services [7]. Women in these settings experience heightened health care needs yet have lower service usage than women in metropolitan areas, including First Nations mothers who face ongoing impacts of colonization and systemic racism within health care [8]. These disparities are compounded by stigma, stoicism, and concerns about confidentiality in close-knit communities, as well as a lack of culturally safe and responsive care [9]. Addressing these inequities requires place-based preventive strategies that respond to local determinants of health and build on existing community infrastructures [10,11]. Preventive approaches are particularly important for reducing downstream service demand, given the limited availability of perinatal mental health treatment in these regions [10,11].

Despite its importance, prevention remains underdeveloped in perinatal mental health care [12]. Treatment alone has not reduced the population burden, and modeling suggests that even full treatment coverage would avert only a minority of cases without concurrent prevention efforts [13]. Evidence-based preventive interventions, including cognitive behavioral and interpersonal approaches, have been demonstrated to reduce perinatal mental illness by up to 40%, generating substantial health care system efficiencies [14]. Yet preventive support rarely reaches women in regional, rural, and remote communities, where access challenges are most pronounced [15].

Digital delivery modes offer a promising avenue for expanding the reach of prevention. Social media platforms, in particular, provide a low-cost, scalable, engaging, and accessible mechanism for reaching regional, rural, and remote populations [13,16]. Women aged 25‐34 years—the modal age of first-time mothers—are among the highest users of platforms such as Facebook (Meta Platforms, Inc) and Instagram (Meta Platforms, Inc) and increasingly seek health information through these channels [17-20]. Social media affordances, including ubiquity, algorithm-driven content personalization, broadcast communication, and the ability to form peer networks, align closely with known preventive mechanisms such as normalization, early psychoeducation, emotional support, and strengthened social connection. These affordances may be especially valuable in regional, rural, and remote communities, where opportunities for connection and professional support are more limited. However, social media also carries risks for perinatal well-being, including exposure to misinformation, information overload, racism, and maladaptive social comparisons [21-23], underscoring the need for careful, evidence-informed, and culturally appropriate intervention design to maximize benefits while mitigating harm [24].

A growing body of evidence supports the effectiveness of social media and digitally delivered interventions for improving mental health outcomes in perinatal populations. Systematic reviews and meta-analyses have demonstrated that internet- and app-based interventions can produce significant reductions in perinatal depression and anxiety symptoms, with effect sizes comparable to those of face-to-face interventions, particularly when they incorporate structured psychological content such as cognitive behavioral therapy or mindfulness-based components [25,26]. Perinatal mental health interventions delivered via social media platforms have shown particular promise, with studies reporting improvements in social connectedness, perceived support, parenting self-efficacy, and reductions in loneliness and depressive symptoms among postpartum women alongside high rates of engagement [27,28]. For example, Facebook-based interventions have reported engagement rates of 83%‐96%, with some also reporting decreased depression severity and improved parenting competence [27,29]. Interventions based on WhatsApp (Meta Platforms, Inc) and WeChat (Tencent Holdings Ltd) have demonstrated feasibility and acceptability in diverse cultural contexts, with some reporting reductions in depression, anxiety, and stress [28,30,31]. Despite this promise, the evidence base remains nascent, with only a handful of studies conducted and critical questions about optimal design and generalizability to underserved populations remaining largely unanswered.

Although interest in social media–based perinatal mental health support is growing, evidence for scalable preventive interventions remains limited. Existing interventions have targeted either prenatal or postpartum women via platforms such as Facebook, WhatsApp, or WeChat to deliver psychoeducation, mindfulness training, peer support, or gamified physical activity programs [27-33]. While most studies report high engagement and improvements in select outcomes, such as parenting competence or mindfulness skills, effects are inconsistent across depression, anxiety, stress, and broader psychosocial functioning [29,31,33]. Most programs are treatment-focused programs for mothers with moderate-to-severe symptoms of depression and/or anxiety rather than universal preventive care [27,28,31,33]. Critically, few interventions have incorporated user perspectives during development or systematically examined the affordances and constraints of social media for prevention. Most have been implemented in high-income urban settings, limiting their generalizability to regional, rural, and remote contexts. To our knowledge, no prior study has conducted a structured needs-affordances analysis to inform the co-design of a social media–based mental health intervention for perinatal women, representing a substantial gap in both research and practice.

This study aimed to address the aforementioned limitation by conducting a novel needs-affordances analysis to support the co-design of a preventive social media–based mental health intervention for women living in regional, rural, and remote Northern Queensland, Australia. Specifically, the study sought to (1) identify the mental health needs of perinatal women in regional, rural, and remote areas and (2) develop prototype design guidelines for a social media program informed by platform-specific affordances and constraints.

## Methods

### Overview

This study represents Phase 1 of a multiphase project that aims to co-design and evaluate a social media–based universal prevention intervention for perinatal mental health in regional, rural, and remote communities, using Northern Queensland, Australia, as an exemplar. The project uses a participatory co-design approach informed by the Double Diamond model [34] through divergent exploration and convergent refinement to develop and evaluate a preventive perinatal mental health intervention program. Specifically, Phase 1 of the project aimed to discover the problems, insights, user needs, and ideas generated by stakeholders and consider how social media may afford or constrain the mental health needs of North Queensland perinatal women, resulting in a set of design principles for the prototype intervention. In Phase 2, these principles will be applied to develop a prototype intervention integrating evidence-based psychological techniques for preventing perinatal depression and anxiety, which will be iteratively refined through additional focus groups and interviews with stakeholders. In Phase 3, the prototype intervention will be evaluated in a pilot trial to assess its feasibility and acceptability with the target population ahead of a future randomized controlled trial.

The research team comprised mental health care researchers and clinicians (a clinical psychologist, a perinatal psychiatrist, a psychologist, a mental health nurse, midwives, and a social worker) as well as a human-computer interaction researcher. A community advisory group of representatives with lived experience of perinatal depression and/or anxiety diagnoses, perinatal mental health clinicians, and local community leaders provided ongoing consultation throughout the design process. The study also adhered to the GRIPP2 (Guidance for Reporting Involvement of Patients and the Public) checklist (Checklist 1) [35].

### Participants

Participants were perinatal women and mental health care professionals residing in Northern Queensland, Australia. Northern Queensland is a regional, rural, and remote area within Queensland, Australia, with a population of approximately 732,000 people spanning over 810,000 km^2^ from Sarina on the central Queensland coast up through Mackay, Townsville, and Cairns to the Torres Strait islands and westwards into inland and remote areas of the Gulf of Carpentaria and Mount Isa [6,36].

Perinatal women were eligible if they (1) were aged ≥18 years, (2) were pregnant or ≤12 months postpartum, (3) resided in Northern Queensland, (4) were proficient in English, (5) did not currently meet criteria for a mental health diagnosis, (6) had prior experience with social media, and (7) did not report moderate/severe symptoms or suicidal ideation on standardized screening measures. Mental health status was assessed using the Edinburgh Postnatal Depression Scale (EPDS), with scores >11 indicating moderate-to-severe distress and endorsement of the suicidality item prompting exclusion and referral to supports [37]. For Aboriginal and Torres Strait Islander mothers, Part 1 of the Kimberley Mum Mood Scale (KMMS) was offered as a culturally appropriate adaptation of the EPDS, with the same scoring and exclusion threshold [38]. Both the EPDS and KMMS are recommended as universal perinatal mental health screening instruments in Queensland Health clinical guidelines, with evidence suggesting approximately two-thirds of women are screened using the EPDS in pregnancy statewide [39,40]. Mothers were also asked whether they had a previous diagnosis of a mental health condition in the demographics survey, although this did not form part of the eligibility criteria. No additional prior mental health data were available for participants.

Mental health care professionals were eligible if (1) they were registered as a mental health care professional, (2) they had experience supporting perinatal mental health, and (3) they lived or worked in Northern Queensland. All participants received an AUD $50 (US $31.48) Prezzee voucher to reimburse them for their time.

Sample size was not predetermined but was guided by the principle of information power, which holds that the adequacy of a qualitative sample is determined by its capacity to illuminate the specific phenomenon under study rather than by a fixed numerical threshold [41]. Information power is shaped by the study aims, the specificity of the participant group, the quality of dialogue, and the analytical approach used. Data collection continued until the research team determined that sufficient information power had been achieved, taking into account the clearly defined study aims, the high specificity of the participant groups, and the depth and richness of data generated across sessions. The final sample of 26 perinatal women and 8 mental health care professionals was considered to reflect this determination.

### Recruitment

Participants were recruited using convenience and community-based sampling methods. Recruitment advertisements were distributed via social media, flyers, community organization newsletters, and media releases inviting women and professionals to “help design a digital program for Northern Queensland women’s perinatal mental health.” Social media marketing was targeted using location and user preference information to reach the intended audience and was used to reach existing users. Snowball recruitment was also encouraged through participants’ social networks.

Interested individuals were directed to an online survey hosted in Qualtrics (Qualtrics International, Inc). After reviewing the participant information sheet, individuals provided informed consent, completed eligibility screening, and answered questions on their demographic information. Participants also provided a preferred nickname for use in group discussions and selected their availability for online or in-person sessions.

### Procedure

Focus groups and individual interviews were conducted from May 6 to July 4, 2025. Eight focus groups (2-4 participants each) and 15 individual interviews were held, with perinatal women and mental health care professionals participating in separate sessions. Sessions lasted approximately 60 minutes on average. Sessions were offered online via Microsoft Teams or in person in Mackay, Townsville, Cairns, and Mt Isa to maximize accessibility.

A semistructured interview guide was developed, consisting of 7 open-ended questions exploring experiences related to perinatal mental health, social support, and digital technologies (Multimedia Appendix 1). Each session began with an acknowledgment of country, an overview of study aims, and a reminder of participant rights, confidentiality, and voluntary participation.

In-person focus groups were cofacilitated by KJL and either ST or JM. Online sessions were facilitated by the lead author (KJL). Sessions lasted 60‐90 minutes, were audio recorded, and were supplemented by facilitator field notes to capture contextual information.

### Data Analysis

#### Analysis of Quantitative Data

Quantitative demographic data were summarized using descriptive statistics (frequencies, percentages, means, and SDs). Current distress levels were characterized using mean scores and SDs on the EPDS and, for First Nations participants, the KMMS [37,38]. Remoteness was classified using the Modified Monash Model (MM1-MM7), and socioeconomic status was indexed using the Socio-Economic Indexes for Areas quintile measure, both based on participants’ postcode [42,43].

#### Analysis of Qualitative Data

##### Overview

A 2-stage needs-affordances analysis was undertaken using a hybrid inductive-deductive approach. Transcript data from perinatal women and mental health care professionals were analyzed separately in the first instance, with each dataset coded independently to preserve the integrity of each group’s perspectives. The resulting themes from both datasets were then brought together in a synthesizing phase, allowing convergences, divergences, and complementarities between lived experience and professional perspectives to be identified and integrated.

##### Stage 1: Identification of Community Mental Health Needs

Transcript data were analyzed inductively using reflexive thematic analysis supported by Taguette (version 1.4.1; The Taguette Project) [44,45]. The lead author (KJL) conducted initial coding of each dataset separately, met regularly with the research team to refine theme boundaries, and consulted with the advisory group to ensure interpretations reflected local context and lived experience. Following independent analysis of each dataset, themes were synthesized across the 2 groups to develop an integrated account of perinatal women’s and professionals’ perspectives on needs, challenges, and priorities relevant to preventive mental health support.

##### Stage 2: Social Media Affordances Analysis

The synthesized needs were then examined deductively using the Moreno and D’Angelo [46] social media affordances framework, which conceptualizes how user needs intersect with functional platform features (eg, connectivity, visibility, social support, and information exchange). This framework provided a structured lens through which to map identified community needs onto specific social media platform capabilities, exploring how social media may enable or constrain each need.

The translational process from needs to prototype design guidelines occurred across 3 complementary steps. First, the focus group and interview guide included questions specifically designed to elicit participant perspectives on social media affordances and preferred intervention features. Participants were asked to reflect on what they believed social media was good and bad for in the context of perinatal mental health and were invited to brainstorm features they would find valuable in a preventive intervention—for example, what would motivate daily engagement, what types of content and features would feel supportive during moments of need, and how they would like local service information to be presented and accessed. This ensured that the affordances analysis was grounded in authentic participant preferences rather than researcher assumptions. Second, the research team applied the affordances framework to systematically analyze how the identified needs could be enabled or constrained by social media platform features, producing a preliminary set of design principles. Third, these preliminary principles were presented to the broader chief investigator team and community advisory group, who were asked to provide input on which features and design elements would best address the identified needs within the regional, rural, and remote context. The finalized prototype design guidelines reflect the integration of participant-generated ideas, affordances-informed analysis, and advisory group input across this iterative process.

##### Reflexivity and Rigor

The study was conducted from a critical realist perspective, recognizing that participants’ accounts reflect both lived experience and broader sociocultural structures, while also acknowledging the influence of researcher positionality [47]. The lead author and facilitator (KJL) was an honors psychology student researcher at the time of data collection, rather than an experienced researcher or clinician, and this positionality is acknowledged as a potential influence on participant rapport and the depth of dialogue in some sessions. KJL also has lived experience of rural health care barriers, which informed her sensitivity to participant perspectives and is reflected in her reflexive memos, maintained throughout the analytical process to document assumptions and decision-making. The presence of mental health care professionals as a separate participant group is additionally acknowledged as a potential influence on power dynamics within those sessions.

Several strategies were used to mitigate these potential influences. In-person focus groups were cofacilitated by experienced mental health care researchers (ST and JM), and KJL received ongoing supervision and methodological guidance from the broader research team throughout data collection and analysis. Perinatal women and mental health care professionals participated in separate focus groups and interviews, ensuring that the potential inhibitory effect of professional authority on mothers’ openness was structurally mitigated. Participants were additionally reminded at the outset of each session of the voluntary nature of their participation, their right to withdraw, and were encouraged to share only what they felt comfortable disclosing.

Coding and theme development were reviewed through iterative team discussions and consultation with the advisory group to support reflexivity and critical engagement with the data, including examination of assumptions, alternative interpretations, and researcher positionality, rather than to achieve coding consensus or interrater reliability [45]. Trustworthiness was supported through verbatim transcription, an audit trail of coding decisions, team-based interpretation to enhance credibility and dependability, and alignment of findings with study objectives to ensure analytic coherence. This approach ensured that the identified needs and design principles were grounded in authentic participant perspectives and were directly applicable to the development of a social media–based preventive intervention.

### Ethical Considerations

Ethics approval was obtained from the James Cook University Human Research Ethics Committee (HREC 24H-9686), and the study was conducted in accordance with the National Statement on Ethical Conduct in Human Research. All participants provided informed electronic consent before completing the demographic survey and attending any focus group or interview session. Consent materials clearly outlined the voluntary nature of participation, the right to withdraw at any time without consequence, and how data would be stored and used. To protect participant privacy and confidentiality, all data were deidentified before analysis. Participants were invited, though not required, to use pseudonyms during focus group sessions, and any identifying information was removed from study records. Sessions were audio recorded solely for transcription, and recordings were destroyed once transcription was complete. No images of individual participants are included in the paper or additional materials. Participants were compensated for their time with an AUD $50 (US $31.48) Prezzee digital gift voucher upon completion of their focus group or interview session.

## Results

### Participant Characteristics

A total of 26 perinatal women participated in the study (Table 1). Of these, 5 were pregnant and 21 were postpartum. Fifteen participated in focus groups and 11 participated in interviews. Participants ranged in age from 25 to 48 years (mean 32.2, SD 4.6 years). Most were multiparous (n=17, 65.4%), partnered (n=24, 92.3%), and living in regional (n=9, 34.6%), rural (n=8, 30.8%), or remote (n=9, 34.6%) areas classified by Modified Monash Model (MM) categories (MM2-MM7). Nearly half (n=12, 46.2%) reported a previous mental health diagnosis, with mean EPDS and KMMS scores indicating low levels of current distress. Most participants used social media daily, primarily Facebook and Instagram, with typical usage between 1 and 3 hours per day.

**Table 1.** Sample characteristics of perinatal women (n=26) across regional, rural, and remote North Queensland.

Characteristic	Perinatal women
Age (years), mean (SD; range)	32.2 (4.6; 25-48)
Perinatal status, n (%)	
Pregnant	5 (19.2)
Postpartum	21 (80.8)
Parity, n (%)	
Primiparity	9 (34.6)
Multiparity	17 (65.4)
Remoteness^a^ (location), n (%)	
Regional (MM2)	9 (34.6)
Rural (MM3-MM5)	8 (30.8)
Remote (MM6-MM7)	9 (34.6)
Area-level socioeconomic disadvantage^b^, n (%)	
0%‐20% (least advantaged)	2 (7.7)
20%‐40%	5 (19.2)
40%‐60%	14 (53.8)
60%‐80%	5 (19.2)
80%‐100% (most advantaged)	0 (0)
Past mental health diagnosis, n (%)	12 (46.2)
Current distress, mean (SD)	
EPDS^c^	5.0 (2.6)
KMMS^d^	7.5^e^
Aboriginal and/or Torres Strait Islander, n (%)	2 (7.7)
Country of birth, n (%)	
Australia	19 (73.1)
Other	7 (26.9)
Spoken language, n (%)	
English speaking	4 (57.1)
Non-English speaking	3 (42.9)
Employment, n (%)	
Full time	3 (11.5)
Part time	4 (15.4)
Unemployed	1 (3.8)
On maternity leave	18 (69.2)
Relationship status, n (%)	
Partnered	24 (92.3)
Single	2 (7.7)
Social media use, n (%)	
≤1 h/d	0 (0)
>1‐3 h/d	17 (65.4)
>3‐5 h/d	7 (26.9)
>5 h/d	2 (7.7)
Preferred social media platforms, n (%)^f^	
Facebook (Meta Platforms, Inc)	18 (69.2)
Instagram (Meta Platforms, Inc)	15 (57.7)
YouTube (Alphabet Inc)	7 (26.9)
TikTok (ByteDance Ltd)	6 (23.1)
Snapchat (Snap Inc)	5 (19.2)
Primary reason for social media use, n (%)^f^	
Connection	23 (88.5)
Information seeking	21 (80.8)
Entertainment	21 (80.8)
Sharing updates	18 (69.2)
Connecting with other mothers	14 (53.8)

a Modified Monash (MM) model classifications [42]: MM1=major cities; MM2=regional centers (>50,000 population); MM3=large rural towns (15,000‐50,000 population); MM4=medium rural towns (5000‐15,000 population); MM5=small rural towns; MM6=remote communities; and MM7=very remote communities.

b Socio-Economic Indexes for Areas (SEIFA) quintiles [43] (postcode-derived).

c EPDS: Edinburgh Postnatal Depression Scale.

d KMMS: Kimberley Mum Mood Scale.

e SD value was not reported for KMMS because only 2 participants were included in this analysis.

f Totals based on participants’ top 3 selections.

Eight mental health care professionals also participated (Table 2). Four engaged in focus groups and 4 in interviews. Participants represented a range of professional backgrounds and had a mean of 16.6 years of clinical experience. They worked across public, private, and community settings in MM2-MM7 regions, and 5 (62.5%) had received specialist perinatal mental health training. Most used social media for less than 1 hour per day, primarily Facebook and Instagram, and reported moderate familiarity with using social media as a psychoeducational tool.

**Table 2.** Sample characteristics of perinatal mental health care professionals (n=8) across regional, rural, and remote North Queensland.

Characteristic	Mental health care professionals
Clinical experience (years), mean (SD; range)	16.6 (7.5; 7-27)
Profession, n (%)	
Counselor	1 (12.5)
Social worker	3 (37.5)
Mental health nurse	2 (25)
Psychologist	1 (12.5)
Occupational therapist	1 (12.5)
Specialist perinatal mental health training, n (%)	5 (62.5)
Remoteness^a^ (location), n (%)	
Regional (MM2)	5 (62.5)
Rural (MM3-MM5)	1 (12.5)
Remote (MM6-MM7)	2 (25)
Area-level socioeconomic disadvantage^b^, n (%)	
0%‐20% (least advantaged)	0 (0)
20%‐40%	2 (25)
40%‐60%	6 (75)
60%‐80%	0 (0)
80%‐100% (most advantaged)	0 (0)
Aboriginal and/or Torres Strait Islander, n (%)	0 (0)
Employment, n (%)	
Nongovernment organization	1 (12.5)
Hospital	1 (12.5)
Community health	3 (37.5)
Private practice	3 (37.5)
Social media use, n (%)	
≤1 h/d	5 (62.5)
>1‐3 h/d	3 (37.5)
>3‐5 h/d	0 (0)
>5 h/d	0 (0)
Preferred social media platforms^c^, n (%)	
Facebook (Meta Platforms, Inc)	5 (62.5)
Instagram (Meta Platforms, Inc)	4 (50)
Pinterest (Pinterest, Inc)	1 (12.5)
WhatsApp (Meta Platforms, Inc)	1 (12.5)
X (formerly Twitter; X Corp)	1 (12.5)
TikTok (ByteDance Ltd)	1 (12.5)
LinkedIn (Microsoft Corporation)	1 (12.5)
YouTube (Alphabet Inc)	1 (12.5)
Familiarity with using social media as a health tool, n (%)	
Not familiar at all	0 (0)
Slightly familiar	2 (25)
Moderately familiar	5 (62.5)
Very familiar	1 (12.5)
Extremely familiar	0 (0)

a Modified Monash (MM) model classifications [42]: MM1=major cities; MM2=regional centers (>50,000 population); MM3=large rural towns (15,000‐50,000 population); MM4=medium rural towns (5000‐15,000 population); MM5=small rural towns; MM6=remote communities; and MM7=very remote communities.

b Socio-Economic Indexes for Areas (SEIFA) quintiles [43] (postcode-derived).

c Totals based on participants’ top 3 selections.

### Stage 1: Needs Assessment via Reflexive Thematic Analysis 

#### Overview

The following 5 themes were identified reflecting the mental health support needs of perinatal women in regional, rural, and remote areas (Table 3): (1) “Women don’t have that proverbial village”—the need for social connection and support, (2) “It can feel like you’re a number”—the need for personalized and respectful health care, (3) “Am I doing this right? Is this normal?”—the need for information that empowers, (4) “We’re the forgotten North”—the need for place-based support, and (5) “Being able to access something on my phone would be so helpful”—the need for accessible digital forms of support.

**Table 3.** Five core mental health needs themes for North Queensland women’s perinatal mental health were derived from reflexive thematic analysis across perinatal women and mental health care professionals.

Theme	Definition	Example code
“Women don’t have that proverbial village”—The need for social connection and support	The need for authentic, consistent relationships with peers, family, and community that provide emotional validation, support, and a sense of belonging.	“If you don’t have any friends yet, because you don’t know anyone and you don’t have family around, it can be very isolating.” (Regional postpartum mother, 29 years) “Especially first time mums, if they don’t yet have that community of other mums, there’s no one to get support from [in terms of] people who are going through the same thing.” (Regional mental health occupational therapist, 39 years).
“It can feel like you’re a number”—The need for personalized and respectful health care	The need for consistent health care interactions that acknowledge and respect the personal values, needs, and preferences of the mother.	“Go along and get help and you’ll feel better...but if you can’t click with them, you’re not going to get anything out of it.” (Regional pregnant mother, 30 years) “It’s just about having a conversation and unfortunately, as a medical profession, we have become very time poor and we don’t have those conversations.” (Regional mental health nurse, 48 years)
“Am I doing this right? Is this normal”—The need for information that empowers	The need for easily accessible, accurate information that equips mothers to make informed decisions and feel self-confident in their role as a mother.	“I definitely think pre and post birth information is really important, so everything from. what to look out for [and] what’s considered normal.” (Remote pregnant mother, 35 years) “How can we develop and deliver this so that we can reach women who may not have previously reached out. it’s all about bridging these gaps, stopping people from falling through the cracks.” (Regional mental health nurse, 48 years)
“We’re the forgotten North”—The need for place-based support	The need for services and resources that are culturally inclusive and safe and provide practical support within the context of regional, rural, remote communities.	“People in North Queensland have less access to things than Brisbane or Melbourne. because of locality and population size...we don’t get as much stuff happening or available to us.” (Regional postpartum mother, 32 years) “I bring that real North Queenslander, blunt approach...there are differences in the demographic and Indigenous health isn’t even on [Brisbane’s] radar.” (Regional social worker, 53 years)
“Being able to access something on my phone would be so helpful”—The need for accessible digital forms of support	The need for digital support tools that are readily accessible, facilitate social connection, and provide relevant information.	“There were definitely gaps in the system...being able to access more information myself would have been really helpful.” (Regional postpartum mother, 28 years) “Everyone uses [social media] so we should definitely infiltrate it with healthcare.” (Regional mental health nurse, 48 years)

#### Theme 1: “Women Don’t Have That Proverbial Village”—The Need for Social Connection and Support 

Mothers described profound loneliness during pregnancy and postpartum, noting that the perinatal period “[took] a big toll on [their] mental health” (regional postpartum mother, 37 years). Many had relocated to Northern Queensland without established social networks and felt geographically and emotionally disconnected from family and friends. Even where partners were present, work commitments or limited understanding left gaps in support. One mother reflected:

*I do not have any family other than my partner’s family here, so that I think having a village is very important.*

[Rural postpartum mother, 38 years]

The need for connection extended beyond family and friends to include peer-to-peer support from other mothers navigating similar experiences. While playgroups and community meetups were helpful, experiences of accessibility, inclusivity, and emotional safety varied. Participants sought nonjudgmental spaces for genuine connection:

*I think that’s just as important for mental health as mental health services postpartum, where you can actually take your baby and meet other women.*

[Regional postpartum mother, 27 years]

Mental health care professionals echoed these concerns, emphasizing the critical role of social connections for well-being: *“*the isolation is huge*”* (regional social worker, 38 years).

#### Theme 2: “It Can Feel Like You’re a Number”—The Need for Personalized and Respectful Health Care* *

Mothers frequently reported depersonalization within health care environments, describing interactions as procedural “box ticking” (remote postpartum mother, 39 years) rather than genuine care, fostering mistrust and disengagement:

*I needed more from them [the healthcare provider]. I needed my own support network to speak up or to help me find that next level of support.*

[Regional postpartum mother, 37 years]

Mothers described feeling pressured to conform to a system that did not reflect their needs or values. Limited access to specialist perinatal mental health care services, inflexible birthing options, and difficulty finding supportive providers left them feeling unseen and disempowered:

*The need to be treated as an individual…not just have to fit into some formula.*

[Rural postpartum mother, 43 years]

Continuity of care, particularly midwife-led models, was viewed as vital for fostering trust and validation. Fragmented care, conflicting advice, and inconsistent provider relationships undermined mothers’ confidence:

*We’re relying on these providers who we drop in and out of to give us helpful advice when they’ve met us for 10 minutes.*

[Rural postpartum mother, 39 years]

Mental health care professionals acknowledged the impacts that this depersonalization has on the mothers’ well-being and the therapeutic outcomes:

*A bad experience with mental health services makes them even more reluctant to engage with us.*

[Regional mental health nurse, 48 years]

#### Theme 3: “Am I Doing This Right? Is This Normal?”—The Need for Information That Empowers* *

Mothers felt unprepared for the emotional and identity shifts of the perinatal period. Challenges such as anxiety, depression, eating disorder symptoms, body dissatisfaction, and birth trauma often went unrecognized until symptoms escalated, with little signposting that it “might [become] a reality” (regional postpartum mother, 25 years). Mental health care professionals acknowledged these challenges, describing the perinatal period as “a real transition” that “requires change” (regional social worker, 66 years). This gap between expectations and reality contributed to feelings of confusion, inadequacy, and shame. First-time mothers described struggling with identity loss in moving from being career-focused individuals to being isolated at home with a newborn:

*Before you’re defined by: you are this person, you work here. Whereas now like, what do I do?*

[Rural postpartum mother, 38 years]

Societal and self-imposed expectations intensified uncertainty, with many questioning if their experiences were “considered normal” and what “doing the right thing” (remote pregnant mother, 35 years) looked like. Partners’ mental health also influenced maternal well-being, yet support for partners was often lacking:

*I didn’t so much have anxiety, but my partner did…another thing that’s not really acknowledged.*

[Remote postpartum mother, 29 years]

#### Theme 4: “We’re the Forgotten North”—The Need for Place-Based Support

Mothers perceived their mental health needs as being overlooked compared to metropolitan areas: 

*[Northern Queensland] sometimes does feel like a different world…at least to the Southeast, it really does.*

[Rural postpartum mother, 28 years]

Mental health care professionals advocated strongly for place-based solutions, noting:

*The local aspect of [the program] is so important…what works for one does not work for all communities.*

[Regional social worker, 30 years]

Both groups emphasized that geographic isolation, workforce shortages, and cultural and social barriers are unique to regional, rural, and remote areas, requiring tailored support that accounts for the diversity within the region. Mothers described systemic inequities, including long waitlists, unaffordable services, and travel burdens:

*City folks don’t understand, they take for granted the ease of access.*

[Remote pregnant mother, 28 years]

Socioeconomic constraints further amplified these barriers:

*I’m on half pay [whilst on maternity leave]…spending money on psychological services is something that we put on the back burner.*

[Rural postpartum mother, 28 years]

Small-town social dynamics also hindered help-seeking, with community expectations to “have everything together” (regional postpartum mother, 28 years) contributing to guilt and shame around mental health needs. Further, culturally and linguistically diverse mothers and First Nations women described perinatal mental health care services as minimal or nonexistent and noted that the dominant Westernized health care model did not always “apply to how [they] think” (regional postpartum mother, 28 years):

*There’s always issues with cultural awareness, sensitivity and safety, whether it’s migrant or Indigenous culture.*

[Rural postpartum mother, 43 years]

#### Theme 5: “Being Able to Access Something on My Phone Would Be So Helpful”—The Need for Accessible Digital Forms of Support*** ***

Participants strongly supported digital mental health interventions, particularly via social media. Mothers envisioned a centralized, curated, “one stop shop” (regional pregnant mother, 30 years) for trusted resources, peer connection, and tailored information:

*A social media platform that allows you to have all those resources in one place would be really good…sometimes you don’t know where to look.*

[Rural postpartum mother, 29 years]

Flexibility, user-controlled content, and moderated peer spaces were considered essential to reduce distress and information overload. Leveraging social media to strengthen in-person community connections was seen as a powerful tool to help prevent perinatal mental health challenges:

*A small community with some meet ups, some social media*—*based check ins would be the way to go.*

[Remote postpartum mother, 39 years]

The mothers had predominantly used Facebook and Instagram previously to access low-level mental health support, praising these platforms for their ease of use and ability to facilitate a sense of autonomy:

*I like to go to a place that is updated regularly, like Instagram for its link in bio feature or Facebook for having info consistently pinned at the top of a page.*

[Remote pregnant mother, 35 years]

Mental health care professionals acknowledged these potential benefits of social media but noted it can be a “mixed bag” (regional psychologist), and particularly emphasized that it cannot replace the therapeutic relationship:

*It won’t be what supports people fully.*

[Regional mental health occupational therapist, 39 years]

### Stage 2: Social Media Affordances Analysis 

#### Overview

The second stage of analysis mapped the identified perinatal mental health needs onto the affordances and constraints of social media, following Moreno and D’Angelo [46], to inform the design of a tailored digital intervention. Table 4 summarizes each need, associated affordances, and design guidelines for the prototype’s content and functionality.

**Table 4.** Mapping identified North Queensland women’s perinatal mental health needs to social media affordances and prototype design guidelines for a future intervention.

Identified need	Applicable affordances	Social media affordances	Social media constraints	Prototype design guidelines
Social connection and support	Social and functional	Peer interaction, shared lived experience, and emotional validation via comments and posts; asynchronous communication allows flexibility: “A person that you can send a message to...you know how you [need to] get it off your chest?” (Rural postpartum mother, 28 years) and “Having a way for women that want to be linked to other women in their community would be helpful.” (Regional Social Worker, 66 years)	Risk of misinformation or online hostility; possible lack of depth in relationships; may reinforce exclusion: “If it’s a page where I’m seeing people getting judged or nasty comments, I’m not going to want to come back to it.” (Remote pregnant mother, 35 years) and “What people write in social media comments can be abusive. that goes on as well.” (Regional Mental Health Occupational Therapist, 39 years)	Moderated peer chat rooms and closed community groups; include peer facilitators; options to join interest-specific pages (eg, postpartum fitness, recipes, and developmental milestones); localized groups based on geography.
Personalized and respectful health care	Identity, emotional, and functional	Ability to tailor content through algorithms or self-selected topics; opportunities for one-on-one messaging or live Q and As^a^: *“*You can choose based on your needs who you’re going to reach out to or what to engage with.” (Regional pregnant mother, 30 years) and “You can choose how much you engage on social media.” (Regional mental health occupational therapist, 39 years)	Risk of generic or impersonal content; no replacement for therapeutic relationships: “I felt like what actually helped the most was those [psychologist] sessions.” (Rural postpartum mother, 29 years) and “I see that as a potential pitfall...you don’t want to get too caught in trying to make it universal.” (Regional social worker, 38 years)	User profiles with optional anonymity; personalized content feeds; features to block or filter topics; daily self-reflection prompts and affirmations.
Information that empowers	Social, cognitive, emotional, and functional	Visual and written content (videos, stories, and infographics) for education; rapid dissemination of evidence-based information: “You want things evidence-based from healthcare professionals...from the nurse, the psychologist, the OT, the physio.” (Rural postpartum mother, 39 years) and “It can grow your confidence rather than just being told that this is what needs to happen.” (Regional mental health occupational therapist, 39 years)	Can promote unrealistic standards; risks reinforcing guilt or shame if not carefully framed: “The negative influence of social media at times when you’ve got people who are eating salads...why can’t I crave a salad?” (Regional pregnant mother, 30 years) and “Other parts of it are not helpful.in terms of unrealistic expectations.” (Regional psychologist, 42 years)	“Ask Anything” sections; content series from health care experts; ability for health care experts to track client progress; partner targeted psychoeducation; library of bite-sized evidence-informed resources; quick links to services; use strengths-based, nonjudgmental language; include diverse representations of motherhood experiences.
Place-based support	Social, cognitive, and functional	Overcomes physical distance; accessible via mobile devices; can highlight regional or cultural content: “If you’re a reader, you might not want to call [a service]...it needs to be culturally and linguistically diverse.” (Regional pregnant mother, 30 years) and “I think one of the strengths is that it can be very focused on local news and local people.” (Regional social worker, 38 years)	Poor internet access in remote areas; dominant cultural narratives often exclude First Nations and migrant voices: “Because of my cultural background, sometimes programs tailored to my local region do not apply to how I think.” (Regional postpartum mother, 28 years) and “We have to take into account the different cultures...make sure we aren’t putting a White Australian English perspective on it.” (Regional mental health occupational therapist, 39 years)	Selectable filters by culture, language, location; localized stories, resources, services, event calendars (eg, playgroups and nurse visits); North Queensland-specific psychoeducation (eg, cyclones and other weather events and travel support); integration of Aboriginal and Torres Strait Islander and culturally and linguistically diverse voices.
Accessible digital formats	Functional	On demand, mobile-first design allows access anytime; no need for in-person appointments: “Telehealth can definitely have its advantages for parents.” (Rural postpartum mother, 39 years) and “I think it’s really good when you have a baby because it can be hard to leave the house.” (Regional psychologist, 42 years)	Digital literacy or affordability may limit access; limited reach to those not on social media: “From a remote perspective, do people have access to computers and smartphones?” (Remote postpartum mother, 29 years) and “People needing a device would be a barrier.” (Regional mental health nurse, 48 years)	Mobile-first, low data design; asynchronous access; integration with free-to-access platforms (eg, Facebook [Meta Platforms, Inc]); inclusive user experience (UX) design for varying digital literacy levels.

a Q and A: questions and answers.

#### Theme 1: Social Connection and Support

Mothers’ desires for social connection align with the social and functional affordances of social media. Peer-to-peer interaction, asynchronous communication, and shared lived experiences can provide emotional validation and flexible engagement. Constraints include the risk of misinformation, online hostility, or shallow relationships. To mitigate these, prototypes could include moderated peer chat rooms, closed community groups with peer facilitators, and interest-specific subgroups (eg, postpartum fitness, local parenting communities):

*Those lived experience stories can be helpful to know that it’s just a season and it will pass.*

[Regional postpartum mother, 35 years]

#### Theme 2: Personalized and Respectful Health Care

The need to feel seen and respected can be supported by identity, emotional, and functional affordances, such as tailored content through self-selected topics or algorithms and one-on-one messaging or live questions and answers. Constraints include generic content and the inability of social media to replace therapeutic relationships. Recommended features include optional anonymity, personalized content feeds, filters, and daily self-reflection prompts:

*I don’t know if there’s scope to add a filter, but I think it [needs to be] a realistic, nonjudgemental space.*

[Regional pregnant mother, 30 years]

#### Theme 3: Information That Empowers

Mothers highlighted gaps in psychoeducation and the need for accessible, evidence-based information. Social, cognitive, emotional, and functional affordances allow rapid dissemination via videos, stories, and infographics. Constraints include reinforcing unrealistic expectations or guilt if the content is poorly framed. Design guidelines include bite-sized, strengths-based resources, expert-led content series, partner-targeted psychoeducation, and diverse representations of motherhood:

*I would love more evidence-based helpful information…[also] to understand how social media can make things worse [rather] than better.*

[Rural postpartum mother, 29 years]

#### Theme 4: Place-Based Support

Social media can address geographic and cultural barriers through social, cognitive, and functional affordances. Online tools allow access without travel or long wait times and can be tailored to local cultural and linguistic needs. Constraints include limited internet access and dominant cultural narratives that may exclude First Nations and migrant voices. Recommended features include localized filters, region-specific resources and events, and culturally inclusive content:

*It has to look like it’s something for me, something for North Queensland…with the pictures and the way it looks.*

[Regional postpartum mother, 25 years]

#### Theme 5: Accessible Digital Formats

The need for flexible, on-demand support maps onto functional affordances, such as asynchronous access, mobile-first design, and replicable content. Constraints include varying digital literacy and affordability. To address this, prototypes should prioritize low-data, mobile-first design, integration with free-to-access platforms, and inclusive user experience design:

*The more barriers you put in front of people, the less likely people are to engage…it needs to be direct links.*

[Regional postpartum mother, 42 years]

## Discussion

### Overview

This study sought to co-design a universal social media–based preventive intervention for perinatal mental illness among women living in regional, rural, and remote Northern Queensland. We identified 5 core mental health needs: social connection and support, personalized and respectful health care, information that empowers, place-based support, and accessible digital formats. Social media shows promise for addressing these needs through peer support, psychoeducation, and culturally responsive content, yet constraints such as misinformation, limited digital literacy, and inequitable access must be addressed in program design. Our findings demonstrate how digital platforms can be leveraged to deliver preventive mental health support in regional, rural, and remote communities, with implications for clinical practice, service design, and public health policy.

### Principal Findings

The first 3 themes mirror the broader perinatal experiences across diverse contexts. Previous literature indicates that mothers value connection and peer support during this period, which is often marked by loneliness [48]. The need for personal and respectful interactions that are clear, evidence-based, and empowering within health care is frequently cited as crucial to perinatal mental health, yet lacking within available supports [48]. Our study extends these findings by demonstrating that these needs are magnified in regional, rural, and remote communities, where social and systemic inequities amplify their salience. While these communities are resilient, this does not deem them exempt from requiring mental health support [9]. Acknowledging and addressing these needs is essential to reducing the compounding effects of inequity and mitigating intergenerational impacts of poor perinatal mental health [4].

Alongside these broader needs, our study highlights the unique systemic and social barriers faced by women in regional, rural, and remote regions. Unlike metropolitan centers, these regions experience workforce shortages that create longer wait times and a lack of individualized care, exacerbated by geographic isolation that has considerable financial and travel-related burdens. The unique social dynamics of smaller communities, characterized by shame, stigma, and a lack of anonymity, created barriers to seeking support beyond that of service availability. Our findings highlight the need for place-based approaches to appropriately address systemic and social inequities and cultural and contextual differences within these communities [11].

Further, our findings suggest that a well-designed social media intervention may help to mitigate some of these challenges. The mothers were supportive of a social media intervention due to its capacity to provide accessible information and act as a “digital village” to foster peer support. This is consistent with previous literature demonstrating that social media is an important source of health information for perinatal women [20]. However, social media cannot address all barriers, including limited digital literacy and structural inequities that remain prominent in these regions. Previous literature demonstrates how social media can perpetuate unhealthy comparison, identity distress, and parenting anxiety, negatively influencing mental health [23]. These concerns were shared by both mothers and mental health care professionals in this study and must be addressed in both the design and functionality of digital platforms. The needs-affordances analysis allowed us to translate these insights into actionable, relevant, and feasible design guidelines. It emphasized the need for a place-based approach that fosters social support while mitigating harmful comparison through moderation. While previous digital health interventions have reduced symptoms of anxiety and depression in perinatal women, the use of social media as a preventive mental health tool remains novel [49]. This research demonstrates that a social media health intervention is a feasible and acceptable platform for delivering preventive, accessible psychoeducational mental health support to perinatal women at a population level.

### Comparison With Prior Work

The needs and design guidelines identified in this study align with prior interventions but add nuance that addresses key gaps. Most previous interventions, including those that were effective in reducing depression and anxiety, incorporated peer connection via chat groups, peer worker support, or forums, highlighting the value of social support [27,30]. However, these interventions were predominantly psychoeducation-based, delivered uniform content, and rarely allowed users to self-direct learning and engagement. Only Hatamleh et al [32] explicitly considered diverse cultural needs, leaving most programs limited in their capacity to engage culturally diverse or regionally isolated mothers [32]. Importantly, none provided options for users to filter or select topics of interest. This was a feature that the mothers in this study identified as key to fostering meaningful engagement and supporting mental health outcomes. Our guidelines directly address these limitations by emphasizing a user-led, autonomous approach grounded in place-based content and design. This approach not only increases the likelihood of engagement and relevance to mothers in regional, rural, and remote communities, but also mitigates the risk of inadvertently excluding or disadvantaging mothers from diverse cultural and linguistic backgrounds. In doing so, these findings extend prior work by illustrating how social media interventions might be tailored to a regional, rural, and remote context to maximize both efficacy and equity, providing actionable guidance for future mental health programs.

The social media intervention could integrate into existing health care systems in a way that alleviates, rather than duplicates, existing burdens. Mental health care services remain predominantly reactive and treatment-oriented, constrained by workforce shortages, increasing health demands, and higher health care costs [50]. In the context of the continuum of care, this program could provide preventive, universal support for perinatal mothers [51]. The potential for a social media intervention to complement existing services was highlighted by the mental health care professionals in this study, which would be particularly beneficial for both their practice and client outcomes. For those experiencing mild distress, peer support and psychoeducation may be sufficient to prevent symptom escalation. The program can also provide a mechanism for mental health screening and referral to formalized supports, complementing existing services and potentially decreasing the need for more intensive services downstream.

### Implications for Policy, Practice, and Future Research

The findings of this study challenge the assumption that digital mental health interventions developed in metropolitan or international contexts can be straightforwardly applied to regional, rural, and remote settings. The mental health needs identified in this study were experienced with a distinctly place-based character, shaped by geographic isolation, workforce shortages, small community social dynamics, and systemic inequity, which generic interventions are unlikely to adequately address. Place-based co-design is, therefore, not merely a methodological preference but a practical necessity for intervention relevance and equity in these communities.

For clinicians and health care services, a well-designed social media intervention holds potential to complement existing perinatal mental health care by providing psychoeducation, peer connection, and normalization of help-seeking at a population level, alongside a mechanism for referral to formalized supports where needed. For policymakers and funders, these findings make a case for sustained investment in co-designed digital mental health infrastructure, including the structural enablers of equitable access such as reliable connectivity, device affordability, and digital literacy support.

More broadly, this study demonstrates that social media is a viable and acceptable intervention platform for adult perinatal women—a population largely absent from the existing evidence base for social media interventions [52]. Our findings highlight the potential for social media to function as an equalizer of access to mental health support, offering an engaging, low-cost, geographically unconstrained, and stigma-reducing touchpoint in settings where structural barriers would otherwise prevent timely care. However, the findings also emphasize that social media is not a neutral delivery channel for mental health support. Realizing this potential requires affordance analysis to be adopted as a standard step in social media intervention development, ensuring platform selection and design are driven by user need.

The next phase of this research is a pilot study involving the development and evaluation of the universal preventive intervention informed by the prototype design guidelines generated here. This pilot phase will use a feasibility and acceptability design, examining whether the intervention can be implemented as intended in regional, rural, and remote communities, and generating preliminary data on outcomes including mental health and well-being, social connectedness, and help-seeking behavior, to inform the design of a subsequent fully powered effectiveness trial. Future studies should also explore the potential for predictive and personalized digital tools, including adaptive algorithms and artificial intelligence, to anticipate psychological distress and triage mothers into appropriate care [53,54].

### Reflection on Patient and Public Involvement

The co-design process generated rich, contextually grounded data and was strengthened by the active involvement of a community advisory group comprising lived experience representatives, clinicians, and local community leaders, who provided ongoing input throughout the research process. The dual online and in-person format was effective in expanding accessibility across a geographically dispersed region, and participants across both formats engaged substantively with the focus group and interview questions, generating detailed and meaningful contributions to the design process.

Notably, the co-design process substantially shaped the direction of the intervention in ways that were not anticipated at the outset. The original intervention concept envisaged a broadcast-style social media presence through which preventive psychotherapeutic content would be disseminated to a largely anonymous audience (eg, a public Instagram or Facebook page). However, the needs-affordances analysis revealed that peer interaction, mutual support, and personalized connection were central priorities for participants, fundamentally reorienting the intervention toward a more interactive and community-driven model. This shift reflects the genuine influence of patient and public involvement on the research outcomes and underscores the value of co-design approaches for intervention development.

However, several challenges were also encountered. Higher-than-anticipated dropout was observed among mothers attending in-person sessions, with cancellations frequently occurring at short notice, consistent with the practical realities of caring for a newborn or infant, including feeding schedules, sleep deprivation, and unpredictable child health. Future co-design research with perinatal populations should anticipate this from the outset, for example, by offering shorter or more flexible session formats or providing childcare support where feasible.

### Strengths and Limitations

This study’s strengths lie largely in its co-design approach, which provided contextually grounded insights supported by lived and clinical experience. Using face-to-face and online methods across diverse regions enhanced depth and representation within the data. The inclusion of a community advisory group with lived experience of perinatal depression and anxiety, alongside a sample in which nearly half of participants reported a prior mental health diagnosis, further strengthened the relevance and authenticity of the findings.

However, several limitations should be noted. First, the sample underrepresented First Nations and LGBTQIA+ (lesbian, gay, bisexual, transgender, queer (or questioning), intersex, and asexual (or agender), and other diverse sexual orientations, gender identities, and sex characteristics) voices, with only 2 participants identifying as Aboriginal and/or Torres Strait Islander despite recruitment through targeted social media advertising, posters in libraries, health care services, community spaces, and media releases. Notably, a number of Aboriginal and Torres Strait Islander mothers expressed interest in participating but were disproportionately screened out at eligibility due to reporting moderate-to-severe symptoms on the KMMS, reflecting the well-documented higher rates of perinatal distress among First Nations mothers in Australia [8,55], and representing an important intersection between our eligibility criteria and First Nations representation. This also highlights a broader tension inherent to universal program co-design: although the intervention is intended to be broadly accessible across diverse communities, the co-design process itself may not have equally captured the perspectives of all subgroups within the target population. First Nations perspectives were nonetheless present at the level of research governance, with Aboriginal and Torres Strait Islander members contributing to both the chief investigator group and community advisory group throughout the study. Future research should prioritize community-embedded recruitment developed in genuine partnership with Aboriginal Community Controlled Health Organizations, employ First Nations researchers and community liaisons, and consider whether supplementary targeted co-design processes are needed to ensure the intervention is culturally safe and relevant for First Nations and LGBTQIA+ communities specifically [56,57].

Second, the exclusion of women currently experiencing moderate-to-severe mental health symptoms or suicidal ideation limits the study’s capacity to capture insights from those at highest risk. This exclusion was intentional and reflects the preventive rather than clinical focus of the intervention; the co-design sample was deliberately aligned with the intended end-user population of the program, which is explicitly preventive and psychoeducational in nature rather than a clinical or therapeutic tool. Accordingly, the findings and design guidelines generated by this study should not be generalized to clinical programs aiming to treat moderate-to-severe perinatal mental health presentations; such programs serve a distinct population with distinct needs and would require their own dedicated co-design processes to determine what women in those contexts want and need from a social media-based intervention. Nevertheless, we acknowledge that women who have experienced symptom escalation may have offered valuable perspectives on what earlier digital support could have prevented. This limitation is partially mitigated by the fact that 46% of participants reported a prior diagnosis of depression and/or anxiety, and by the inclusion of lived experience representatives of perinatal depression and/or anxiety on the advisory group. Future work could consider engaging women with current or recent clinical presentations as a distinct consultative group to complement the perspectives captured here.

Third, the eligibility criterion requiring prior social media experience was included to ensure participants could engage meaningfully with the affordances analysis. While this criterion could theoretically filter out the most digitally excluded members of the target population, a tension worth acknowledging given that digital literacy and affordability emerged as barriers in our findings (Theme 5), no participants were excluded on this basis in practice. This reflects the near ubiquity of social media use even among populations with limited digital literacy or constrained financial resources. Recruitment through both online and offline channels further reduced the likelihood that digitally excluded women were unable to learn about or express interest in the study. Nonetheless, future evaluation phases should monitor whether the intervention is equally accessible and acceptable to women with lower levels of digital literacy to ensure equitable reach.

Fourth, social media affordances are not static and may shift over time as platform designs, algorithms, community norms, privacy policies, and access conditions evolve. The design principles generated by this study reflect the affordances landscape at the time of data collection, and future intervention development and evaluation phases will need to remain responsive to changes in the social media environment to ensure continued relevance and safety.

Fifth, recruitment via convenience sampling and snowball methods, including social media advertising and participant referral networks, may have preferentially engaged women who are already digitally active and connected to community networks. Women with lower digital literacy, limited internet connectivity, or reduced engagement with community organizations, including those in the most geographically remote parts of Northern Queensland, may therefore be underrepresented. Future co-design and implementation research should incorporate targeted, community-embedded recruitment strategies to reach these subgroups.

### Conclusion 

This study aimed to identify the mental health needs of perinatal women in regional, rural, and remote communities and to co-design a framework for a preventive social media–based intervention informed by platform-specific affordances and constraints. Using a participatory co-design approach with 26 perinatal women and 8 mental health care professionals in Northern Queensland, Australia, 5 core mental health needs were identified: social connection and support, personalized and respectful health care, information that empowers, place-based and culturally safe support, and accessible low-burden digital formats. A needs-affordances analysis demonstrated that social media holds promise for addressing these needs through peer support, psychoeducation, normalization, and timely information delivery, provided that known constraints are proactively addressed in program design, including misinformation, privacy risks, harmful social comparison, and inequitable digital access. These findings were synthesized into prototype design guidelines to inform subsequent phases of intervention development. Taken together, these findings address a significant gap in the literature and offer a replicable methodological approach for co-designing digital mental health interventions with underserved perinatal populations.

## Supplementary material

10.2196/91778 Multimedia Appendix 1 Focus group or interview session outline.

10.2196/91778 Checklist 1 GRIPP2 checklist.

## Abbreviations

EPDS: Edinburgh Postnatal Depression Scale
GRIPP2: Guidance for Reporting Involvement of Patients and the Public
KMMS: Kimberley Mum Mood Scale
LGBTQIA+: lesbian, gay, bisexual, transgender, queer (or questioning), intersex, and asexual (or agender), and other diverse sexual orientations, gender identities, and sex characteristics

## References

[1] Howard LM, Khalifeh H (2020). Perinatal mental health: a review of progress and challenges. World Psychiatry.

[2] Law S, Ormel I, Babinski S (2021). Dread and solace: talking about perinatal mental health. Int J Mental Health Nurs.

[3] Khajehei M (2015). Mental health of perinatal women. WJOG.

[4] Rogers A, Obst S, Teague SJ (2020). Association between maternal perinatal depression and anxiety and child and adolescent development: a meta-analysis. JAMA Pediatr.

[5] (2026). The Australian health system. Australian Government Department of Health, Disability and Ageing.

[6] (2025). Annual report 2024-2025. https://annualreport2025.nqphn.com.au/.

[7] Galbally M, Watson SJ, Coleman M (2023). Rurality as a predictor of perinatal mental health and well‐being in an Australian cohort. Australian J Rural Health.

[8] Fox H, Topp SM, Lindsay D, Callander E (2021). Ethnic, socio‐economic and geographic inequities in maternal health service coverage in Australia. Health Plan Manag.

[9] Kavanagh BE, Corney KB, Beks H, Williams LJ, Quirk SE, Versace VL (2023). A scoping review of the barriers and facilitators to accessing and utilising mental health services across regional, rural, and remote Australia. BMC Health Serv Res.

[10] Arango C, Díaz-Caneja CM, McGorry PD (2018). Preventive strategies for mental health. Lancet Psychiatry.

[11] Klepac B, Mowle A, Riley T, Craike M (2023). Government, governance, and place-based approaches: lessons from and for public policy. Health Res Policy Sys.

[12] Fitzgerald L, McNab S, Njau P (2024). Beyond survival: prioritizing the unmet mental health needs of pregnant and postpartum women and their caregivers. PLOS Glob Public Health.

[13] Patel V, Saxena S, Lund C (2018). The Lancet Commission on global mental health and sustainable development. Lancet.

[14] O’Connor E, Senger CA, Henninger ML, Coppola E, Gaynes BN (2019). Interventions to prevent perinatal depression: evidence report and systematic review for the US Preventive Services Task Force. JAMA.

[15] Lewis Johnson TE, Clare CA, Johnson JE, Simon MA (2020). Preventing perinatal depression now: a call to action. J Womens Health (Larchmt).

[16] Perkins D, Farmer J, Salvador‐Carulla L, Dalton H, Luscombe G (2019). The Orange Declaration on rural and remote mental health. Australian J Rural Health.

[17] Petkovic J, Duench S, Trawin J (2021). Behavioural interventions delivered through interactive social media for health behaviour change, health outcomes, and health equity in the adult population. Cochrane Database Syst Rev.

[18] Kemp S (2021). Digital 2021: Australia. Data Reportal.

[19] (2025). Australia’s mothers and babies. Australian Institute of Health and Welfare.

[20] Gow ML, Henderson M, Henry A, Roberts L, Roth H (2024). Australian postpartum women want reputable health information delivered via social networking sites. Aust NZ J Obst Gynaeco.

[21] Li B, Jin N, Wang Y, Hou X, Meng J, Zhang Y (2025). Perinatal women’s perception of maternal health information quality on digital media: scoping review. J Med Internet Res.

[22] Tate MK (2023). The impact of social comparison via social media on maternal mental health, within the context of the intensive mothering ideology: a scoping review of the literature. Issues Ment Health Nurs.

[23] Smith M, Mitchell AS, Townsend ML, Herbert JS (2020). The relationship between digital media use during pregnancy, maternal psychological wellbeing, and maternal-fetal attachment. PLoS One.

[24] Li SH, Achilles MR, Spanos S, Habak S, Werner-Seidler A, O’Dea B (2022). A cognitive behavioural therapy smartphone app for adolescent depression and anxiety: co-design of ClearlyMe. tCBT.

[25] Ansaari N, Rajan SK, Kuruveettissery S (2025). Efficacy of in-person versus digital mental health interventions for postpartum depression: meta-analysis of randomized controlled trials. J Reprod Infant Psychol.

[26] Xie H, Cong S, Wang R (2024). Effect of eHealth interventions on perinatal depression: a meta-analysis. J Affect Disord.

[27] Boyd RC, Price J, Mogul M, Yates T, Guevara JP (2019). Pilot RCT of a social media parenting intervention for postpartum mothers with depression symptoms. J Reprod Infant Psychol.

[28] Yang M, Jia G, Sun S, Ye C, Zhang R, Yu X (2019). Effects of an online mindfulness intervention focusing on attention monitoring and acceptance in pregnant women: a randomized controlled trial. J Midwife Womens Health.

[29] Kernot J, Lewis L, Olds T, Maher C (2019). Effectiveness of a Facebook-delivered physical activity intervention for postpartum women: a randomized controlled trial. J Phys Act Health.

[30] Shorey S, Chee CYI, Ng ED, Lau Y, Dennis CL, Chan YH (2019). Evaluation of a technology-based peer-support intervention program for preventing postnatal depression (part 1): randomized controlled trial. J Med Internet Res.

[31] Zhang X, Lin P, Sun J (2023). Prenatal stress self-help mindfulness intervention via social media: a randomized controlled trial. J Ment Health.

[32] Hatamleh R, AbdelMahdi AbuAbed AS, Abujilban S, Joseph R (2023). Using social media platforms to enhance the delivery of a childbirth education program. J Perinat Neonatal Nurs.

[33] Guevara JP, Morales K, Mandell D (2023). Social media-based parenting program for women with postpartum depressive symptoms: an RCT. Pediatrics.

[34] (2005). The Double Diamond. Design Council.

[35] Staniszewska S, Brett J, Simera I (2017). GRIPP2 reporting checklists: tools to improve reporting of patient and public involvement in research. BMJ.

[36] (2025). Annual report 2024-2025. https://cdn.prod.website-files.com/6498e7a6f8c93e238042358e/69265b6c903c1183f3e4b33e_WQPHN%20FY25%20Annual%20report%20FINAL%20DIGITAL%20SINGLES.pdf.

[37] Cox JL, Holden JM, Sagovsky R (1987). Detection of postnatal depression. Development of the 10-item Edinburgh Postnatal Depression Scale. Br J Psychiatry.

[38] Carlin E, Ferrari K, Spry EP, Williams M, Atkinson D, Marley JV (2022). Implementation of the ‘Kimberley Mum’s Mood Scale’ across primary health care services in the Kimberley region of Western Australia: a mixed methods assessment. PLoS One.

[39] San Martin Porter MA, Betts K, Kisely S, Pecoraro G, Alati R (2019). Screening for perinatal depression and predictors of underscreening: findings of the Born in Queensland study. Med J Aust.

[40] (2024). Queensland clinical guidelines. Queensland Health.

[41] Malterud K, Siersma VD, Guassora AD (2016). Sample size in qualitative interview studies: guided by information power. Qual Health Res.

[42] (2025). Modified Monash Model. Australian Government Department of Health and Aged Care.

[43] (2011). 2033.0.55.001 - Census of Population and Housing: Socio-Economic Indexes for Areas (SEIFA), Australia, 2011. https://www.abs.gov.au/ausstats/abs@.nsf/DetailsPage/2033.0.55.0012011.

[44] Rampin R, Rampin V (2021). Taguette: open-source qualitative data analysis. JOSS.

[45] Braun V, Clarke V (2019). Reflecting on reflexive thematic analysis. Qual Res Sport Exerc Health.

[46] Moreno MA, D’Angelo J (2019). Social media intervention design: applying an affordances framework. J Med Internet Res.

[47] Braun V, Clarke V (2021). Thematic Analysis: A Practical Guide.

[48] Schwartz H, McCusker J, Law S, Zelkowitz P, Somera J, Singh S (2021). Perinatal mental healthcare needs among women at a community hospital. J Obstet Gynaecol Can.

[49] Chan KL, Chen M (2019). Effects of social media and mobile health apps on pregnancy care: meta-analysis. JMIR Mhealth Uhealth.

[50] Dixit SK, Sambasivan M (2018). A review of the Australian healthcare system: a policy perspective. SAGE Open Med.

[51] Baker E, August K, Mangra N (2025). The impact of a continuum of care on social determinants of health and health disparities: an IEEE perspective. THMT.

[52] Zhang Q, Huang Z, Sui Y (2025). Social-media-based mental health interventions: meta-analysis of randomized controlled trials. J Med Internet Res.

[53] Barrett M, Boyne J, Brandts J (2019). Artificial intelligence supported patient self-care in chronic heart failure: a paradigm shift from reactive to predictive, preventive and personalised care. EPMA J.

[54] Shatte ABR, Hutchinson DM, Teague SJ (2019). Machine learning in mental health: a scoping review of methods and applications. Psychol Med.

[55] Owais S, Faltyn M, Johnson AVD (2020). The perinatal mental health of indigenous women: a systematic review and meta-analysis. Can J Psychiatry.

[56] Escobar-Viera CG, Melcher EM, Miller RS (2021). A systematic review of the engagement with social media-delivered interventions for improving health outcomes among sexual and gender minorities. Internet Interv.

[57] Reilly R, Stephens J, Micklem J (2020). Use and uptake of web-based therapeutic interventions amongst Indigenous populations in Australia, New Zealand, the United States of America and Canada: a scoping review. Syst Rev.

