# Supplementary material for: A Preventive Social Media Intervention for Perinatal Depression and Anxiety in Regional, Rural, and Remote Communities: Participatory Co-Design Study
Source: J Med Internet Res. 2026 Jun 10;28:e91778. doi: 10.2196/91778 (PMC13252985; doi:10.2196/91778)
Supplement: Multimedia Appendix 1 [file jmir-v28-e91778-s001.docx]

**Focus group/interview session outline**

**‘Discover’ Focus Group Semi-Structured Guide**

**BEGIN RECORDING**

**Welcome and introductions.**

**Welcome & Overview**

1. Acknowledgement of Country
2. Thanks for coming along to today’s group chat/interview. My name is Kacey Lynch, I am a 4th year psychology student currently undergoing my thesis project (and I’m here with Dr Sam Teague and Dr Jessica Muller, we’re both mental health researchers at James Cook University). Your time and willingness to share your experiences are truly appreciated.

**Brief Intro to Study**

1. Today’s session is about listening to your voices and experiences to help us shape a social media-based support that genuinely meets your needs. By the end of today, we hope to understand your experiences and needs, as well as any barriers you might face in accessing mental health support. This will help us ensure that whatever we create is useful and supportive for women in your community.

**Participation Guidelines**

A few things to remember before we start:

- ***Respect and Confidentiality***: What’s shared in this group stays within this group. We ask that everyone respects each other’s stories and experiences, and we won’t share anything you say outside this group unless it’s as part of a summary, without names or identifying details.
- ***Voluntary Sharing***: You’re welcome to share as much or as little as you feel comfortable with. You can skip any questions that you’d rather not answer. We want this to feel like a safe space for everyone.
- ***Take turns sharing ideas***: Since each of you brings your own unique experiences, we’ll go one at a time to make sure everyone has a chance to share. Feel free to share whatever’s on your mind—we’re here to listen.

Does anyone have any questions? Well, let's begin with a bit of background on how we got to this point!

- Gap in services in North Queensland, barriers to accessing mental health support
- Social media has significant reach across the Australian population

**Rapport-Building**

To start, let’s go around and introduce ourselves. You could tell us your name and share one thing you enjoy about your daily life—like your favourite way to start the day. There’s no pressure; it can be as simple or as detailed as you like.

**Questions:**

1. To start, can you share a little about your experiences with mental health during pregnancy and postpartum? This can be your own experience, or if you’d like, feel free to speak more generally about things you’ve noticed in the community. (~10min)
2. What would you say are some of the biggest mental health needs for women during this period? What kinds of support or resources do you think would make the most difference? (~10min)
3. What challenges have you or others faced in accessing mental health support? Are there specific barriers related to location, culture, or services that make it harder to get help? (~10min)
4. What would make you want to check in with a social media page for perinatal mental health every day? What types of features (e.g., daily check-ins, expert advice, personal stories) would keep you engaged? (~10min)
5. Think about a time when you really needed support—what would have helped in that moment? If this social media page was part of your daily routine, what would you want to see first when you open it? (~10min)
6. If the platform could connect you to local services, what are the most important ones to include? How would you like this information to be presented so it’s easy to find and use? (~10min)
7. Do you think it’s important that a program like this is tailored to your local region? Why or why not? (10min)

**Wrap up**

Thank you all for sharing such important experiences and ideas. Let me summarize some of the main themes we’ve discussed. [Summarise briefly.] Is there anything else you’d like to add or clarify?

Your insights today have given us a strong foundation. In our next session, we’ll focus on designing the actual social media support tool, brainstorming ideas, and looking at design options together. We’d love for you to join us again if you’re interested.

Thank you again for your time and trust in sharing with us. If you’d like, we’d be happy to keep you updated on the progress and share opportunities to review and refine the design further.
