# Supplementary material for: A Preventive Social Media Intervention for Perinatal Depression and Anxiety in Regional, Rural, and Remote Communities: Participatory Co-Design Study
Source: J Med Internet Res. 2026 Jun 10;28:e91778. doi: 10.2196/91778 (PMC13252985; doi:10.2196/91778)
Supplement: Checklist 1 [file jmir-v28-e91778-s002.docx]

GRIPP-2 (Guidance for Reporting Involvement of Patients and the Public) reporting checklist.

| **Section and topic** | **Item** | **Reported on page no.** |
| --- | --- | --- |
| 1. Aim | Report the aim of PPI study | 2, 8 |
| 1. Methods | Provide a clear description of methods used for PPI study | 9-16 |
| 1. Study results | Outcomes: Report the results of the PPI study, including both positive and negative outcomes | 16-33 |
| 1. Discussion and conclusion | Outcomes: Comment on the extent to which PPI influenced the study overall. Describe positive and negative effects | 34-43 |
| 1. Reflections/critical perspective | Comment critically on the PPI in the study, reflecting on things that went well and those that did not, so others can learn from the experience | 38-39 |
